# Cerebral embolism complicating left atrial myxoma: a case report

**DOI:** 10.11604/pamj.2016.24.140.9778

**Published:** 2016-06-14

**Authors:** Zairi Ihsen, Mssaad Hela, Mzoughi Khadija, Jnifene Zouhayer

**Affiliations:** 1 Department of Cardiology, Habib Thameur Public Hospital, Bab El Fallah, Tunis, Tunisia; 2 Department of Pediatric Cardiology, La Rabta Hospital, Tunis, Tunisia

**Keywords:** Cardiac tumor, surgery, pediatric, atrium

## Abstract

Cardiac myxoma are the most common benign primary cardiac tumors that can lead to many complications as described in literature. Here we report the case of a boy aged 11 that was referred for etiological diagnosis of ischemic stroke. Transthoracic echocardiography reveals a myxoma in the left atrium. Patient was referred to surgery. The diagnosis was confirmed and the mass was completely resected.

## Introduction

Cardiac myxoma are the most common benign primary cardiac tumors that can lead to many complications as described in literature. They can mimic mitral stenosis, infective endocarditis and other vascular disease [1]. Atrial myxomas are found in approximately 14-20% of the population and can lead to embolization, intra cardiac obstructions, conduction disturbances and lethal valve obstructions [2]. Atrial myxomas are associated with systemic embolization in 30 to 40% of cases. Here we report the case of a boy aged 11, that was referred for etiological diagnosis of ischemic stroke. Transthoracic echocardiography reveals a myxoma in the left atrium. Patient was referred to surgery. The diagnosis was confirmed and the mass was completely removed.

## Patient and observation

A boy of 11 who was sent by the pediatrician for an etiological investigation of ischemic stroke. Physical examination revealed blood pressure 99/70 mmHg. Heart rhythm was regular at 70 bpm. The patient had no prior history of heart murmurs, syncope, shortness of breath, or chest pain. Further physical examination revealed a soft grade 2/6 systolic murmur at the left sternal border, with no diastolic murmur present.

**Figure 1:**
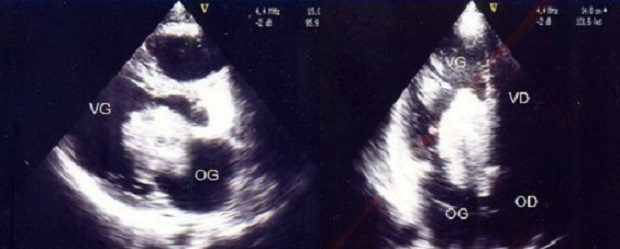
Echography showing the left atrial myxoma. At left: Parasternal long axis, at right: 4 chambers view

**Figure 2:**
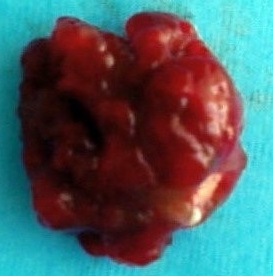
Myxoma with cauliflower appearance

A transthoracic echocardiogram was performed that revealed a large tumor occupying the left atrium, very mobile, measuring 5cm long strongly mimicking a myxoma (Figure 1). The patient was referred to surgery. After median sternotomy and opening of the left atrium, it was found a myxomatous huge mass with a cauliflower appearance was protrusion outside the cavity, it was multilobed, very friable and pedicle. It measures 5 cm long axis It was attached to the interatrial septum with small base of implantation of 0.5 cm diameter. The mass was completely resected, taking based implementation (Figure 2).

The immediate postoperative course was uneventful. Cardiac ultrasound control revealed a mild mitral regurgitation, which has not progressed with a decline of 15 years (since 1993).

## Discussion

Atrial myxomas represent approximately 50% of all cardiac tumors, occurring mainly in the 3rd–6th decade of life [3]. They appear mainly from the left atrium subendocardial mesenchymal cells. Although they are histologically benign, they may be lethal because of their strategic position. They can mimic every cardiac disease and malignant processes. Symptoms include cardiac failure due to obstructed filling causing dyspnea, pulmonary edema, and signs of right heart failure. It may lead to syncope, systemic embolism or sudden death. It's estimated that atrial myxomas cause up to 0.5% of ischemic strokes [4].

Embolization to the central nervous system may result in transient ischemic attack, stroke, or seizure. Strokes are often recurrent [5] and may be embolic or hemorrhagic, the presentation ranging from progressive multi-infarct dementia to massive embolic stroke causing death. Transthoracic echocardiography has a sensitivity of around 90% in detection of left atrial myxoma; the sensitivity of transesophageal examination is even higher [6].

It was described that myxomatous emboli frequently leads to formation of cerebral aneurysms and may lead to severe neurologic complications such as intracerebral hemorrhage [7].

## Conclusion

2D echocardiography provides substantial advantages in detecting intra-cardiac tumors. Although atrial myxomas are usually benign or asymptomatic, there is the possibility of diastolic embolization. Since surgical excision has been reported to alleviate symptoms associated with cardiac myxomas, early identification and removal is preferable.
